# Simulated Resuscitation in a Psychiatric Setting (SRiPS)

**DOI:** 10.1192/bjo.2025.10433

**Published:** 2025-06-20

**Authors:** Abigail Runicles, Leopold Rudolph, Boris Warszawski, Rebecca Howard

**Affiliations:** South London and Maudsley NHS Trust, London, United Kingdom

## Abstract

**Aims:** Resuscitation events can be highly stressful, particularly for those expected to lead them. As a psychiatric doctor on-call, you are often the most senior member of the resuscitation team. However, the available equipment and expertise differ from the medical settings trainees may be accustomed to. This project aimed to create a safe and supportive environment for on-call doctors to practice leading emergency resuscitation scenarios using the available equipment in a psychiatric setting. The goal was to better equip doctors to provide optimal patient care in real emergencies.

**Methods:** Doctors on the on-call rota across three Oxleas sites were invited to participate in a simulated resuscitation event. Before the session, a questionnaire was distributed to assess their baseline knowledge and confidence regarding resuscitation.

The session was led by an Advanced Life Support (ALS) trainer, a resuscitation officer, and a core trainee. Trainees engaged in three on-call scenarios, including a ligature emergency. Each scenario was followed by a structured debrief, and the sessions were recorded for review.

After the event, participants completed a follow-up questionnaire to evaluate changes in their confidence and knowledge. In total, 11 doctors at various training stages attended the sessions at Green Parks House and Oxleas House.

**Results:** 27% of the cohort had no prior experience with Advanced Life Support (ALS). Before the session, only 9% of doctors strongly agreed with the statement: “I know what is expected of me in a cardiac arrest scenario.” After the session, this increased to 82%.

Following the training, 91% of doctors became familiar with the contents of the emergency medical bag. Before the session, only 36% of participants somewhat agreed that they felt confident leading a cardiac arrest, with none strongly agreeing. After the session, 82% of participants reported increased confidence, including 18% who strongly agreed.

Additionally, 100% of participants found the session beneficial and stated they would recommend it to a colleague.

**Conclusion:** Overall, the session provided a valuable introduction to resuscitation in a psychiatric setting. Nearly all participants reported improved confidence, increased knowledge of their role in a cardiac arrest scenario, and greater awareness of the contents of the red emergency bag and blue drug box.

